# Graphene-Reinforced Carbon-Bonded Coarse-Grained Refractories

**DOI:** 10.3390/ma15010186

**Published:** 2021-12-27

**Authors:** Enrico Storti, Jens Fruhstorfer, Bruno Luchini, Adéla Jiříčková, Ondřej Jankovský, Christos Georgios Aneziris

**Affiliations:** 1 Institute of Ceramics, Refractories and Composite Materials, TU Bergakademie Freiberg, Agricolastr. 17, 09599 Freiberg, Germany; ondrej.jankovsky@vscht.cz (O.J.); aneziris@ikfvw.tu-freiberg.de (C.G.A.); 2 Chair of Ceramics, Montanuniversität Leoben, Peter-Tunner-Str. 5, 8700 Leoben, Austria; jens.fruhstorfer@unileoben.ac.at; 3 Tata Steel Netherlands, R&D Ironmaking, Steelmaking and Casting Materials Engineering & Mathematical Modelling, 1970 CA IJmuiden, The Netherlands; bruno.luchini@tatasteeleurope.com; 4 Department of Inorganic Chemistry, University of Chemistry and Technology Prague, Technická 5, 16628 Prague 6, Czech Republic; adela.jirickova@vscht.cz

**Keywords:** graphene oxide, thermal shock resistance, packing density, refractories, composites

## Abstract

Carbon-bonded alumina refractories offer excellent thermal shock performance but are lacking in terms of mechanical strength. In the present contribution, the influence of the particle packing and the addition of graphene oxide (GO) to carbon-bonded alumina refractories on the physical and mechanical properties before and after thermal shock was investigated. Coarse tabular alumina grains were coated by a GO suspension and used to prepare dry-pressed compacts. The included graphite fraction (15 wt%) was either regarded as a lubricating matrix component or as a quasi-spherical component of a calculated density-optimized aggregate size distribution. During coking, the GO was reduced to thermally reduced graphene. The porosity, true density and thermal shock behavior in terms of the cold modulus of rupture (CMOR) and Young’s modulus were compared. Samples with a higher density were obtained when the irregularly shaped graphite was considered as the matrix component (lubricant). The results showed that the use of GO had a positive impact on the mechanical properties of the graphene-reinforced Al_2_O_3_–C refractories, especially in the case of a less optimized packing, due to the bridging of delamination gaps. In addition, the thermal shock only had a minor impact on the Young’s modulus and CMOR values of the samples. SEM investigation revealed very similar microstructures in coked as well as thermally shocked samples.

## 1. Introduction

Carbon-bonded alumina refractories (Al_2_O_3_–C) are key ceramic materials in the steel industry for their extraordinary chemical, mechanical and thermal properties. Due to the nature of the steelmaking process, materials in contact with the molten metal often require a high thermal shock resistance, a high corrosion and erosion resistance, a low wettability and a sufficient mechanical strength at high temperatures [1,2,3,4,5]. Compared to pure oxide refractories, the implementation of a carbon bond and the addition of graphite may improve all these properties [1,6].

In the case of carbon-bonded alumina, refractories with approximately 30 wt% residual carbon after coking are state-of-the-art for functional components, such as submerged entry nozzles, monobloc stoppers and ladle shrouds in steel casting operations [7]. However, the content of carbon in refractories ought to be decreased in order to lower the CO_2_ emissions as well as a possible carbon contamination of the molten metal [8,9].

Fruhstorfer et al. [10,11] demonstrated that it is possible to retain a low wettability and extraordinary corrosion resistance and thermal shock performance with only ≈3% of residual carbon amount. On the other hand, the mechanical properties of the same carbon-bonded refractories decreased unsatisfactorily. Therefore, the strengthening of low-carbon Al_2_O_3_–C refractories still remains an important research topic.

In the case of a carbon-bonded refractory, the microstructure (in coked state) is characterized by ceramic grains with a specific grain size distribution (aggregates), graphite flakes, voids (pores and cracks) and kept together by a carbonaceous partly-graphitized bonding phase (matrix) that is formed during curing and coking from resin or pitch components [12,13]. Due to differential expansion and shrinkage during the thermal treatment, delamination gaps and microcracks develop around the grains and flakes within the carbonaceous matrix, which limit the mechanical strength but are also responsible for the excellent flexibility and thermal shock performance [14,15].

In order to strengthen a refractory with a low amount of carbon, an optimization of the grain size distribution and an addition of reinforcing components bridging the delamination gaps—connecting aggregates and matrix—seems beneficial. Other possibilities are, e.g., the particle shape engineering (toughening by contact shielding [16]) or the implementation of transformation toughening mechanisms (zone shielding [16]). The effect of the grain size distribution was mainly shown for carbon-free refractories. For the densest packing, the amount of grain contact points increases, which contributes to an increased structural strength [17]. However, an increased strength commonly correlates with a higher strength drop during thermal shock [18,19].

The use of various nano-scaled carbon materials as reinforcement in refractories has been reported in the literature. It was proven that boron- and nitrogen-doped expanded graphite effectively reinforced Al_2_O_3_–C refractories [20]. Interestingly, nickel-loaded ultrafine microcrystalline graphite powders can facilitate the in situ formation of ceramic whiskers, leading to increased densification and mechanical properties of low carbon Al_2_O_3_–C materials [21,22].

Boron carbide has the ability to reduce the brittleness of Al_2_O_3_–C refractories, while promoting the in situ growth of MWCNTs after the pyrolysis of resin binder [23]. The presence of nickel nitrate catalyst in the matrix of Al_2_O_3_–C refractories can help to form in situ MWCNTs and SiC whiskers, which contributed to a higher cold modulus of rupture in comparison to refractories without such a catalyst [24]. Alumina nanoparticles have effectively been used to reinforce ultra-low carbon MgO refractories by the formation of magnesium aluminate spinel at 1500 °C [25].

Mechanical properties and oxidation resistance of Al_2_O_3_–C refractories can be significantly improved by the co-addition of CNTs and polycarbosilane. The presence of polycarbosilane prevented the agglomeration of CNTs [26]. Mertke and Aneziris reported that the combination of alumina nanosheets and carbon nanotubes together with semiconductive silicon improved the thermal shock resistance of carbon-bonded alumina [27]. Alumina-coated graphite was also used as replacement for expanded graphite [28]. The authors observed that the alumina coating delayed the oxidation of graphite, and the products delivered better thermal shock resistance than common Al_2_O_3_–C refractories.

Another promising group of nanoscaled additives is graphene oxides and graphite oxides, which are intensively studied materials due to their outstanding properties [29]. Graphite oxide can be easily prepared by the oxidation of graphite in a highly acidic environment using strong oxidizing agents, such as potassium chlorate or potassium permanganate [30,31,32,33,34]. Among the production methods, Tour’s method provides the highest amount of oxygen-containing functional groups within graphene sheets [33,34]. It was also demonstrated that even higher amounts of oxygen functionalities can be achieved by multiple oxidation steps [35,36].

Graphene oxide (GO) can be easily prepared from graphite oxide by its exfoliation, e.g., by ultrasonication. This material was recently used in the synthesis of filters, membranes and for water treatment [37,38]. Graphene or graphite oxides are also important starting materials in the synthesis of other graphene derivatives [39,40]. Zhang et al. [41] fabricated a multilayer graphene nanosheets/ZrB_2_–SiC ceramic composite by in situ thermal reduction of graphene oxide. The addition of 5 vol% graphene improved the fracture toughness of ZrB_2_–SiC by 75 %, and the flexural strength was increased by 100 %.

The crack deflection of the graphene-reinforced composite was promoted by graphene crack bridging and pull-out. Al_2_O_3_–C refractories containing GO and additives of Al, Si and SiO_2_ were also presented. The GO enhanced the formation of ceramic whiskers due to their higher reactivity in comparison to graphite flakes. The mechanical properties of these Al_2_O_3_–C composites, such as the cold modulus of rupture were investigated [42]. GO was similarly used to increase the mechanical properties of MgO–C refractories [43].

The aforementioned studies implemented different strengthening mechanisms but none acting directly on the gaps and their structure-weakening effect. Furthermore, to the authors’ knowledge, the implementation of irregularly shaped flake graphite into particle size distributions has not been addressed yet. In the present work, the effect of a graphene oxide coating of the alumina aggregates on the mechanical properties of Al_2_O_3_–C refractories was investigated. In this case, the graphene oxide should act directly in the delamination gap and connect the oxide particles with the carbonaceous matrix. In addition, in terms of the particle size distribution, the aim was to assess whether the graphite belongs best to the aggregates or to the matrix. For all compositions, the amount of graphite was limited to 15 wt%, representing an approximate carbon reduction of 50% compared to state-of-the-art carbon-bonded alumina refractories.

## 2. Materials and Methods

The physical and mechanical properties before and after a single thermal shock (1TS) of dry-pressed compacts were determined in order to investigate the reinforcing effect of graphene oxide-coated coarse aggregates and the effect of including the graphite fraction in a calculated aggregate size distribution (ASD).

### 2.1. Materials

The used oxidic raw materials were tabular alumina (T60/64, Almatis GmbH, Ludwigshafen am Rhein, Germany) fractions up to a maximum grain size $d_{\max}$ of 3 mm. The particle size distributions and true densities required for the batch calculation are given elsewhere [44].

Furthermore, in line with Roungos and Aneziris [45], fine natural graphite (AF 96–97, Graphit Kropfmühl AG, Hauzenberg, Germany) and coarse natural flake graphite (NFL 92–94, Graphit Kropfmühl AG, Hauzenberg, Germany) were used. The particle size distributions (cf. Table 1) were measured by laser granulometry (Beckman Coulter LS 230) according to the European Standard DIN EN 725-5 after a careful deagglomeration treatment supported by ethanol as a deflocculant in an ultrasonic bath for 5 min.

Due to the plate-like shape of graphite, however, the true particle size distributions in terms of volume-equivalent particle sizes are supposedly different from the measured ones. Nevertheless, for strongly irregularly-shaped particles the sphere of influence is also increasing [46] and the measured distributions might be applicable for density-optimized batches. The true density of graphite was reported to be 2.22 g/cm^3^ [47,48]. For the carbon bond, novolac-type phenolic resins (Momentive Specialty Chemicals, Waterford, NY, USA) were used in liquid (PF 6662 FL) and powder (PF 0235 DP) form together with a curing agent (Hexamethylene tetramine, Momentive Specialty Chemicals, Waterford, NY, USA) as described by several studies [11,45].

The graphene oxide suspension was prepared according to the modified Tour’s method [33,49]. The mixture of concentrated sulfuric acid and phosphoric acid in a volume ratio 9:1 (360 mL:40 mL) was cooled to 5 °C. Next, we added graphite (3.0 g) and subsequently potassium permanganate (18.0 g). The ongoing reaction heated the mixture itself to ∼20 °C due to the exothermic process. The reaction mixture was stirred and then heated up to 50 °C for 1 h.

Next, the mixture was cooled to 20 °C and poured onto 500 g of ice. After the ice dissolved, 30% hydrogen peroxide was added (50 mL) to remove the remaining unreacted potassium permanganate and manganese dioxide. The obtained GO was then purified by repeated centrifugation and redispersion in deionized water, until a negative reaction on sulphate ions with Ba(NO_3_)_2_ was achieved. After centrifugation, the final GO slurry contained ∼5 g of graphene oxide and 200 mL of water, which corresponds to a concentration of 25 g/L.

### 2.2. GO Characterization

Scanning transmission electron microscopy (STEM) was performed by means of a Tescan Lyra dual beam microscope, equipped with a field emission gun as an electron source and a STEM sample holder. To perform the measurements, the GO suspension was drop cast on a 200 mesh Cu grid for transmission electron microscopy and dried inside a vacuum oven at 50 °C. STEM measurements were carried out using a 30 kV electron beam. Elemental composition and mapping were performed using an energy-dispersive spectroscopy (EDS) analyzer (X-MaxN) with a 20 mm^2^ silicon drift detector (Oxford instruments) and AZtecEnergy software. Before these measurements, the samples were placed on a carbon conductive tape.

Scanning electron microscopy and energy-dispersive spectroscopy measurements were carried out using a 10 kV electron beam. Furthermore, combustible elemental analysis (CHNS-O) was performed using a PE 2400 Series II CHNS/O Analyzer (Perkin Elmer, Waltham, MA, USA). The instrument was used in operating mode for detection of carbon, hydrogen and nitrogen (the most robust and interference-free mode) in order to convert the sample elements to simple gases (CO_2_, H_2_O and N_2_). The PE 2400 analyzer automatically performed combustion, reduction, homogenization of gaseous products, separation and detection.

A MX5 microscale (Mettler Toledo) was used for precise weighing of the samples (1.5–2.5 mg per single sample analysis). Using this procedure, the accuracy of CHN determination was better than 0.30% abs. The internal calibration was performed using N-fenyl urea. High resolution X-ray photoelectron spectroscopy (XPS) was performed using an ESCAProbeP spectrometer (Omicron Nanotechnology Ltd., Taunusstein, Germany) with a monochromatic aluminum X-ray radiation source (1486.7 eV). Wide-scan detection of all elements was performed. Relative sensitivity factors were used to evaluate the carbon-to-oxygen (C/O) ratios from the obtained spectra.

The dried sample was placed on a conductive carrier made from a high purity silver bar. An electron gun was used to eliminate sample charging during the measurement (1–5 V). Fourier transform infrared spectroscopy (FT-IR) measurements were performed on a iS50R FTIR spectrometer (Thermo Scientific, Waltham, MA, USA). The measurement was performed with a diamond ATR crystal, DLaTGS detector and a KBr beamsplitter in the range 4000–400 cm^−1^ at a resolution of 4 cm^−1^. The measurement was performed in reflectance mode using Smart SAGA reflectance accessory, MCTD* detector and a KBr beamsplitter.

Before the measurement, the sample was prepared by drying the dispersion on a gold-coated silicon substrate. An inVia Raman microscope (Renishaw, Wotton-under-Edge, UK) in backscattering geometry with CCD detector was used for Raman spectroscopy. DPSS laser (532 nm, 50 mW) with applied power of 5 mW and 50× magnification objective was used for the measurement. Instrument calibration was achieved with a silicon reference, which gives a peak position at 520 cm^−1^ and a resolution < 1 cm^−1^. The samples were suspended in deionized water (1 mg/mL) and ultrasonicated for 1 min.

The suspension was then deposited on a small piece of silicon wafer and dried. X-ray powder diffraction (XRD) was carried out using a Bruker D2 Phaser (Bruker AXS GmbH, Karlsruhe, Germany), a powder diffractometer with Bragg–Brentano geometry, applying CuKα radiation (λ = 0.15418 nm, U = 30 kV, I = 10 mA) and rotation of 5 rounds per minute. The step size was set to 0.02025° (2θ), and the whole dataset was acquired in the angular range of 5–80°.

### 2.3. Coating Procedure

For the graphene-reinforced batches, in addition to the as-supplied raw materials, the 1–3 mm tabular alumina fraction was coated by graphene oxide (GO). The grains were coated with a highly viscous dispersion of GO in water (see above) using a simple dip-coating device developed in-house. Coated particles were then dried in air atmosphere at 60 °C for 48 h. From weights (before and after the coating), it was determined that the sample of coated coarse grain alumina contained approximately 0.4 wt% of GO, which was a reasonable amount for our further experiments.

As the actual thickness of the coating consisted of a few atomic layers, no additional particle size distribution and true density analyses were performed for the coated aggregates. Instead, these were taken as direct replacement of the uncoated particle fraction in the reference batch. Nevertheless, the effectiveness of the coating procedure was investigated by scanning electron microscopy (SEM) (ESEM XL30FEG, FEI Company, Hillsboro, OR, USA).

### 2.4. Batch Design

The batches were then designed according to a modified Andreasen model, referred to as Ψ model [50]. The model is shown in Equation (1) where CPFT(*d*) is the cumulative volume percentage of particles with diameters ≤d. In contrast to the standard Andreasen model [51], in the modified approach, a linear distribution modulus function dependent on the particle size and the parameters $n_{\min}$ and $n_{\max}$ is applied. The parameter $n_{\min}$ characterizes the distribution modulus of an infinitely small particle size and $n_{\max}$ the one at the maximum grain size. Therefore, the amounts of fine and coarse particles can be adjusted separately by the minimum and maximum distribution moduli, $n_{\min}$ and $n_{\max}$, respectively.

(1) $$CPFT\left(d\right)=100\%\times {\left(\frac{d}{d_{\max}}\right)}^{n_{\min}+d\times \frac{n_{\max}-n_{\min}}{d_{\max}}}$$

Parameters were chosen to maximize the bulk density with $n_{\min}=0.2$ and $n_{\max}=0.6$ [18]. Furthermore, the graphite content was reduced to 15 wt% in all batches. As it was not clear whether the graphite would act like a grain (i.e., taking a larger sphere of influence due to its irregular grain shape) or if it would behave more like a lubricant (by bending around the alumina grains), two batches were calculated: one including the graphite in the aggregate size distribution (ASD) calculation of the batch and the other excluding it from the calculation.

The results from the latter were then multiplied by 0.85 to account for the 15% graphite to add. In line with Roungos and Aneziris [45,52], the powder and liquid resins were introduced in 4 and 2 wt% amounts, respectively, and the curing agent was used with a content of 10% based on the total resin amount. The final batches (including/excluding graphite in the calculation of the aggregate size distribution (ASD); GO-coated/uncoated coarsest alumina fraction) are shown in Table 2.

### 2.5. Sample Preparation

The batches were prepared by means of ordered mixing using a conventional mortar mixer (ToniMIX, Toni Baustoffprüfsysteme GmbH, Berlin, Germany). First, the coarse grains larger than 0.5 mm were dry-mixed for 1 min. Next, the liquid resin was added and mixed for 5 min. Finally, the finer grain fractions and remaining additives were added. The batch was mixed again for 3 min, then the walls of the mixing container were scraped, and the mixture was further stirred for 7 min [11].

Seven bars (150 × 25 × 25 mm^3^) of each composition were pseudo two-side pressed on a uniaxial press (ES 270, RUCKS Maschinenbau GmbH, Glauchau, Germany). To avoid possible cracks caused by entrapped air, the chamber was de-aired twice at 1/3 and 2/3 of the maximum pressure of 100 MPa [11].

Next, the pressed samples were cured at 180 °C as described by Brachhold et al. [47]. The samples were then heat treated inside retorts filled with calcined petcoke (to approach reducing conditions). The heating curve used a 3 K/min ramp up to the maximum temperature of 1000 °C, which was held for 5 h before free cooling [47]. During the treatment, the GO was reduced to thermally reduced graphene: the oxygen functionalities decomposed into water, carbon monoxide and carbon dioxide.

### 2.6. Sample Characterization

After coking, the bulk density of the pressed sample bars was determined by weighing and measuring the dimensions. In addition, the Young’s modulus was estimated in compliance with the European Standard DIN EN 843-2 by measuring the ultrasonic runtime (BP-700 Portable Ultrasonic Tester, UltraTest GmbH, Achim, Germany) three times per sample on all samples of every batch. For the calculation of the Young’s modulus, a constant Poisson’s ratio of 0.2 was assumed.

On three bars per batch, then, the cold modulus of rupture (CMOR) was measured by three-point bending, according to the European Standard DIN EN 993-6 with the aid of a testing machine (TIRAtest 2420, TIRA GmbH, Schalkau, Germany). The true and bulk density were also determined on the broken pieces by He-pycnometry (Accupyc 1330, Micromeritics GmbH, Unterschleißheim, Germany) and by toluene immersion according to the European Standards DIN 66433/ISO 15901-1 and DIN EN 993-1, respectively.

The remaining samples were quenched once (1TS) from the temperature of 950 °C, using compressed air in accordance to the European Standard DIN EN 993-11. In order to evaluate the damage caused by the thermal shock, the Young’s modulus, and then the CMOR were measured and compared to the values from the unquenched samples. In addition, scanning electron microscopy coupled with EDS (ESEM XL30, FEI/Philips, Mainz, Germany) was used. Finally, the true and bulk density were determined on the remaining broken pieces.

## 3. Results

In this section, the characterization of graphene oxide (GO) and the coating of aggregates by GO are first presented. Next, the bulk density, true density, total porosity, CMOR, Young’s modulus, and the effects of thermal shocking on those properties are presented. Two designs of batches were investigated: the first one including the graphite within the calculation of the aggregate size distribution (AC), and the second one excluding graphite from the calculation (A). Both batches were prepared either with graphene oxide-coated coarse alumina grains (AC–GO and A–GO) or with uncoated coarse grains (AC and A).

Statistical tests were applied on the data in order to detect and confirm the trends and observations. Specifically, analyses of variances (ANOVA) were used on all results to investigate the influence of individual factors and of their interactions. An ANOVA is an extension of the t-test, where two variables and their difference can be tested. The tests were performed with the statistic software “R” [53,54]. The means are considered significantly different when the test’s *p* value is ≤0.05.

### 3.1. Go and Coating Characterization

**Figure 1:**
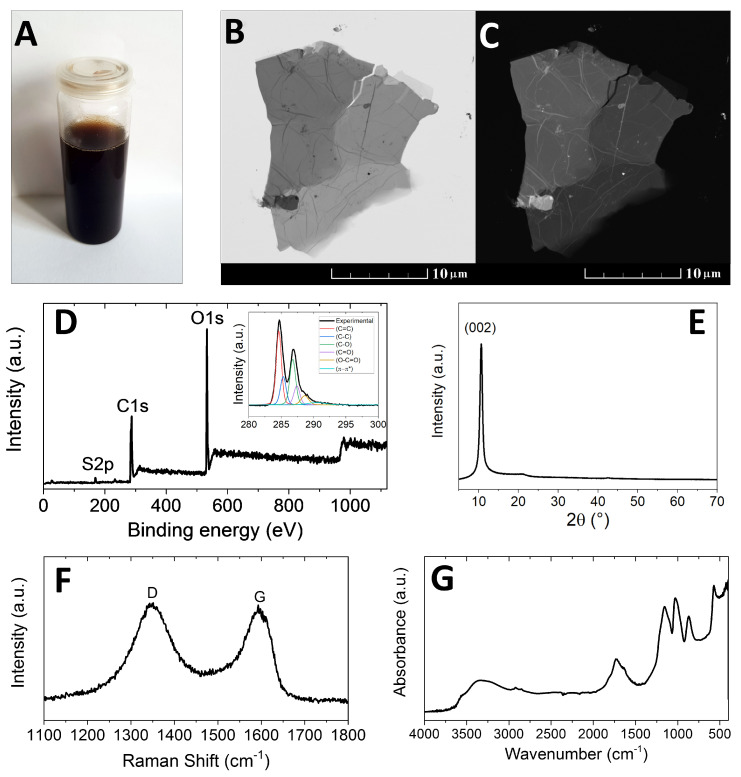
(**A**) GO suspension, (**B**) STEM image of GO in bright field mode, (**C**) STEM image of GO in dark field mode, (**D**) XPS survey spectrum and C1s detail, (**E**) XRD analysis of GO, (**F**) Raman spectroscopy of GO and (**G**) FT-IR analysis of GO.

A photograph of the GO suspension in water after sonication is shown in Figure 1A. The suspension had a brown colour, which is typical for highly oxidized graphene oxides where permanganates are used as oxidizing agents. For the analysis of the chemical composition, a small part of the GO slurry was dried in a vacuum oven at 50 °C. The morphology was studied using STEM (see Figure 1B,C). The micrographs show graphene oxide with a few layers and the typical wrinkled structure.

Figure 1D shows the XPS survey spectrum, where the two major peaks correspond to C1s and O1s. In addition, a weak S2p peak was also detected: the sulphur impurity present in the sample derived from the sulphuric acid used for the synthesis and possibly also from the starting graphite. XPS cannot detect hydrogen; however, the twinning C1s peak was deconvoluted in order to quantitatively characterize the six different bonding states of carbon: C–C (284.5 eV); C=C (285.2 eV); C–O (286.2 eV); C=O (287.8 eV); O–C=O (289.0 eV); and π–π* interactions (291.0 eV). The following quantities of each bonding state were observed: 38.6% of C–C; 14.1% of C=C; 26.0% of C–O; 12.7% of C=O; 5.6% of O–C=O; and 3.0% of π–π* interaction (see the inset of Figure 1D).

The deconvolution confirmed the presence of oxygen-containing functionalities (carboxyls, carbonyls, ketones, epoxides and hydroxyls). The calculated composition was 69.5 at.% of C, 29.6 at.% of O and 0.9 at.% of S. These results were confirmed by means of EDS, which gave the following results: 67.8 at.% of C, 31.0 at.% of O and 1.2 at.% of S (average of 5 point measurements). A higher oxygen content was obtained by elemental analysis, that yielded the following: 36.4 at.% of C, 34.1 at.% of O, 28.4 at.% of H and 1.1 at.% of S. This method is important for the determination of the hydrogen content, while the estimation of oxygen is not as precise as with other methods. The XRD analysis (see Figure 1E) was used in order to determine the interlayer distance within graphene oxide.

In contrast with pure graphite with (002) reflection around 26.3° and interlayer distance of 3.3 Å, the reflection (002) of GO was found at 10.6° revealing an interlayer distance of 8.4 Å. Raman spectroscopy showed two local maxima (Figure 1F). The first local maximum at 1343 cm^−1^ (D band) represented carbon with sp3 hybridization. The second local maximum at 1593 cm^−1^ (G band) is related to the sp2 hybridization—regular graphene lattice. The D/G ratio of 1.03 indicated high levels of oxidation/defectivity in the sample.

Using FT-IR spectroscopy (see Figure 1G), at around 3400 cm^−1^ a broad O–H stretching band was visible, which originated from hydroxyl groups. The C=O stretching band was found at around 1730 cm^−1^. The stretching band of C–O was detected at 1160 and 1030 cm^−1^. The C–O stretching band suggested a high content of both hydroxyl and carboxyl groups.

**Figure 2:**
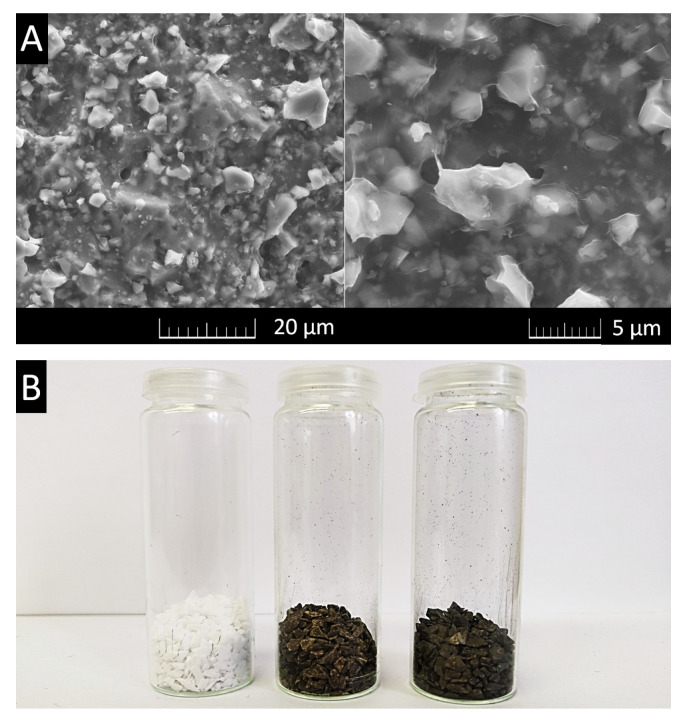
(**A**) SEM images of alumina grains coated with graphene oxide in two magnitudes and (**B**) uncoated grains (**left**), coated grains (**middle**) and treated grains (**right**).

SEM was used to verify that the alumina grains were effectively coated. In Figure 2A, a very thin GO film is clearly visible on the surface of the alumina grains, proving the high effectiveness of the coating process. Small gaps between the alumina grains and the carbonaceous matrix could be filled with GO, achieving a pseudo-binding effect with the ability to increase the strength of the material. Photographs of uncoated, coated and treated grains are displayed in Figure 2B. The coated grains had a typical brown layer of graphene oxide on the top, while the thermally reduced form of GO on the treated grains was black.

### 3.2. Samples Characterization and Effects of Particle Size Distribution, GO Coating and Thermal Shock

Table 3 presents the results of the measurements regarding the physical properties before and after thermal shock. For the coked state, the bulk densities were determined by measuring the dimensions and weight of the bars or by toluene immersion. In the first case, the applied ANOVA returned that the bulk density was significantly influenced by both main factors (addition of GO & consideration of graphite in ASD calculation) and their interaction, with *p* values ≤ 0.049. The bulk densities decreased when GO was added to the batch and when graphite was included in the aggregate size distribution calculation, giving in return for batch A the highest bulk density in coked state.

This indicates that graphite is better regarded as a lubricant in the matrix than as a quasi-spheric particle in the ASD. Regarding the GO effect, it can be noted that during coking of the bars, gases like CO, CO_2_ and H_2_O evolve from the GO, leaving small pores in the bars. Additionally, it was tested if the bulk densities measured by dimensions and masses and by immersion tests differed. For the coked state, however, the test returned that the bulk density results did not depend significantly on the analysis method (p>0.94).

For the bulk densities determined by immersion tests, of the factors GO addition, graphite consideration and thermal shock, only the GO addition had a (statistically) significant effect with p<0.045. The *p* values for the main effects of the graphite consideration and the thermal shocking were <0.086, marking “tendencies” in terms of statistics. The *p* values were supposedly higher due to the higher standard deviations returned by the immersion technique compared to the other measurement method. All two factor and three factor interaction effects were not significant, where the third factor was the thermal shocking. In addition to the effects discussed before, during thermal shocking either developing microcracks or chemical changes could cause a decreasing bulk density.

Changes of the true density (cf. Table 3) would suggest changes in the chemical composition of the material [55]. The ANOVA returned that of the main factors GO addition, graphite consideration and thermal shock, only the latter seemed to have an effect. However, there should be no significant change of true density with thermal shock cycling in case of carbon-bonded alumina. If the graphite was partly consumed by the oxidation during heating inside the furnace, the true density of the remaining material would actually increase (the true density of graphite is lower than that of the alumina grains). In this case, the chemical composition probably did not change significantly, thus, the obtained values can be considered the same before and after thermal shock.

More interesting, however, was that the graphite consideration and GO addition interacted negatively (in the two-factor as well as three-factor interaction), meaning that extreme values resulted if one factor was on the lower and the other on the upper level. The graphite consideration is associated with a lower bulk density, higher porosity and thus a higher possibility of oxidation and burnout of the graphite. This means that a denser packing reduced the burnout. This effect occurred in particular when no graphene oxide was added. With the addition of graphene oxide, the consideration of graphite in the packing (lower bulk density) resulted in a lower true density, whereas a denser structure led to a higher true density. This effect could be explained by the fact that for a denser structure also the reaction of graphene is inhibited.

**Figure 3:**
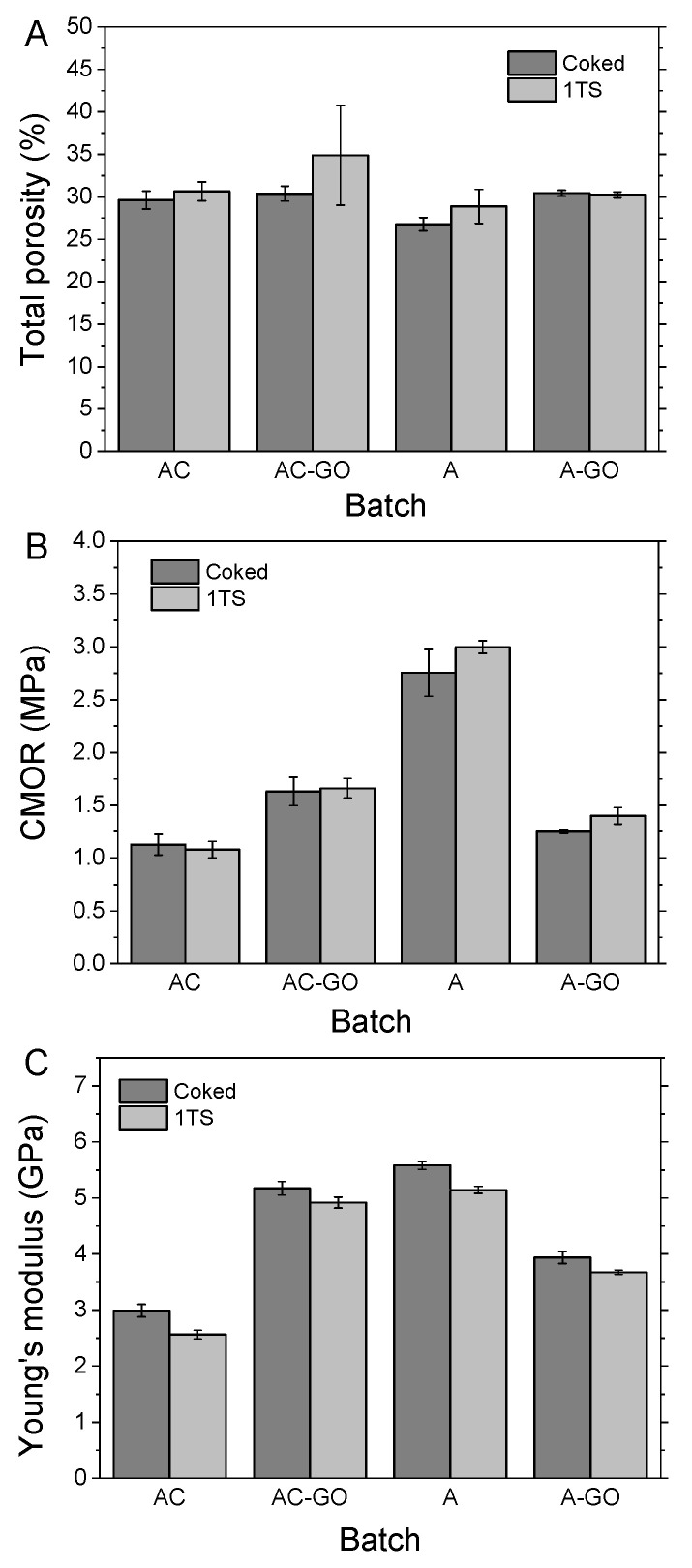
The means and standard deviations of total porosity (**A**), CMOR (**B**) and Young’s modulus (**C**) before (coked) and after (1TS) thermal shock. A = graphite not included in the aggregate size distribution calculation; AC = graphite included in calculation; –GO = coarse alumina coated with graphene oxide.

It can be noted that the closed porosity (calculated as total porosity − open porosity) was not influenced statistically by any factor or interaction. On the other hand, the factors (GO addition; graphite consideration; thermal shock) and interactions thereof influenced both the open and total porosities (≡1 − bulk density/true density) similarly. The total porosities are also presented as bar diagram in Figure 3A. The factor GO addition influenced open and total porosity significantly (p<0.05). Interestingly, thermal shocking had no significant influence under any test. Similar results were reported by Storti et al. who investigated the influence of different fiber additives on the properties of cement-free alumina castables [56].

The effect of the GO addition on the open and total porosity was expectedly reciprocal to its effect on the bulk density. The high value of total porosity of GO-containing samples was caused by the process of gas release from GO during thermal treatment, which is in good agreement with the low bulk density. As already discussed for the bulk densities, excluding the graphite from the aggregate size distribution calculation and considering it as a lubricant in the matrix gave a denser material with lower total porosity. Moreover, the interaction of both effects led to very low values of open porosity for the batch A (without GO and excluding the graphite from the ASD calculation).

Regarding the cold modulus of rupture (CMOR), it has to be noted that this is the key property when improving the strength of carbon-bonded alumina refractories with a low amount of carbon. The results of the three-point bending tests are presented in Figure 3B. The applied ANOVA indicated very high significances for the main factors GO addition and graphite consideration, as well as for their two-factor interaction (*p* values <2 × $10^{-7}$). Surprisingly, the factor thermal shocking again had no significant influence on the CMOR, which can be also observed in Figure 3B when considering the standard deviations.

This observation is quite remarkable, since the CMOR of carbon-bonded alumina refractories typically drops by about 30% after the first thermal shock cycle due to microcracking [47,48,52]. A possible explanation is related to the interstitial voids, which likely developed in smaller sizes but with higher numbers. This would, in turn, improve the thermal shock performance [57,58]. The possibility of surface oxidation of the refractory bars while holding at 950 °C inside the furnace (right before thermal shock) was also considered.

However, decarbonization of the samples in terms of a color brightening was not visible on the surfaces or from the cross sections. The loss of carbon from graphite or graphene (with a lower coefficient of thermal expansion compared to alumina) would likely result in thermal tensile stresses on the surface. Such stresses would in turn decrease the CMOR of refractory samples, which was not observed in the present work.

It is evident that, without GO addition and the exclusion of graphite from the ASD calculation, higher strengths were obtained. In particular, the interaction effect reveals that batch A had the highest CMOR values before and after thermal shock. However, high strengths were also obtained when GO was added to a batch where graphite was considered in the calculation (AC–GO). On the one hand, a better packing with lower porosity gives an increased strength and at the same time the matrix components have the possibility to form a stronger bond. On the other hand, when the packing was not optimal (when graphite was included in the ASD calculation), the graphene had the chance to provide a reinforcing effect.

Regarding the contribution of reduced graphene oxide, a chemical bonding with the alumina can be excluded. On the other hand, an interaction with the carbon matrix during thermal treatment by reduced graphene is possible. However, the main contribution of this additive should be mechanical. The strengthening effect can be attributed to a microstructural effect, such as interlocking and bridging of the delamination gaps around the oxide particles.

A possible reason why, in the case of better packing, the GO addition resulted in lower strength (A–GO) might be that, in the denser structure, the gas release from the GO during coking led to internal stresses, causing microcracking in addition to generating pores. A similar effect was reported for the carbon bond by Luchini et al. [15]. In a less dense structure (AC–GO), the developed stresses were smaller and did probably not cause cracking apart from leaving void space.

The measurement of the Young’s modulus *E* is typically more sensitive than a three-point bending test. The obtained values are presented in Figure 3C. The ANOVA applied on these results returned that only the GO addition, the graphite consideration and their two-factor interaction had significant effects (p<0.0011) but not the thermal shocking. In line with the results for the CMOR, including graphite in the ASD calculation (AC) resulted in lower values of *E*. Similarly, the GO addition led to an increase of *E* when the packing was not optimal (AC–GO).

Comparing Figure 3B,C, the main difference is that batch A had much higher CMOR values than *E* values in relation to the other batches, but still the highest Young’s modulus. Therefore it is not surprising that the interaction for *E* performed similarly to that for the CMOR with the highest *E* values resulting for batch A, followed by AC–GO.

Since the same samples could be tested before and after thermal shock (non-destructive technique), it was possible to calculate the drop of *E* with thermal shock for each sample and to average the results, cf. Table 3. An ANOVA with the factors of GO addition and graphite consideration was applied on the *E* drop. It resulted that the graphite consideration in tendency (p=0.0505), the GO addition and their interaction significantly (p<0.005) influenced the drop of *E* with thermal shocking. This means that a drop of the mechanical properties was proven, although the drops were smaller than the deviations within the batches themselves.

Nevertheless, those low drops are not typical for carbon-bonded alumina refractories, for which the decrease is usually in the order of 20% [59]. The drop of *E* was lower when graphite was excluded from the calculation of the ASD and if GO was added. The interaction was positive, giving the highest value of the *E* drop for the batch AC. A strongly disrupted microstructure would only marginally degrade during thermal shock. The exclusion of graphite from the calculation gave a denser packing, possibly with smaller voids in higher number. Interpreting this as a more disrupted microstructure despite the higher density could explain the first behavior. The addition of GO led to a gas release during coking causing microcracking, delivering lower CMOR and *E* but also a lower *E* drop.

**Figure 4:**
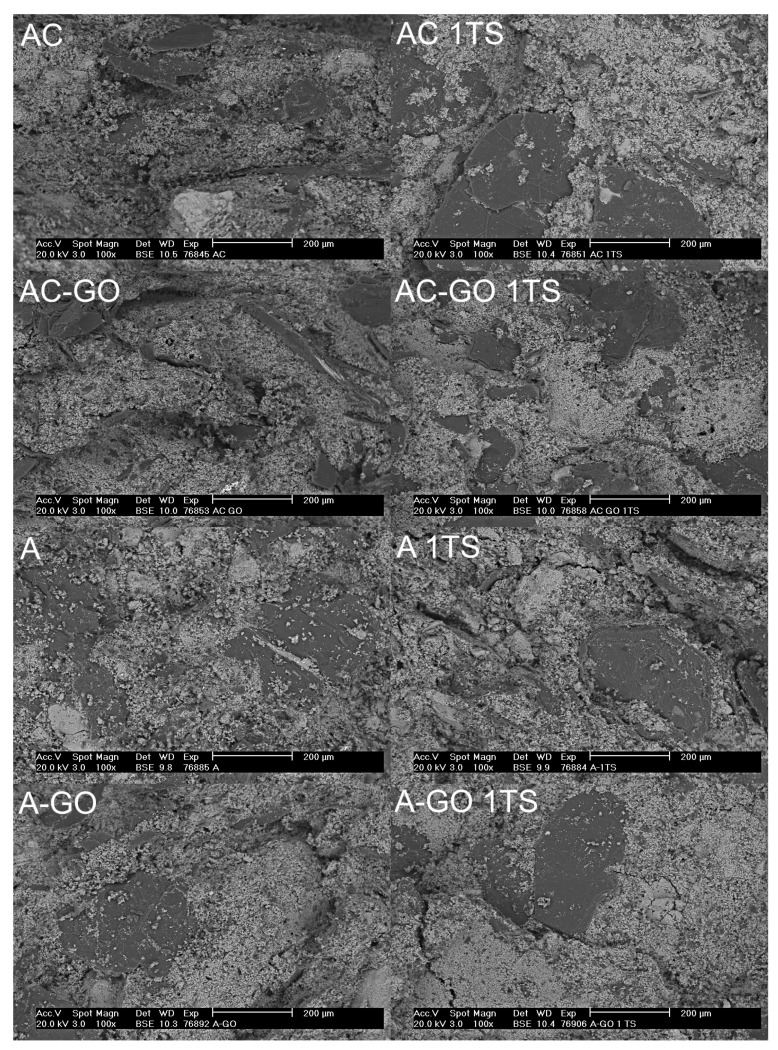
Scanning electron micrographs of the fracture surface of refractory samples, before and after (1TS) one thermal shock cycle. A = graphite not included in the aggregate size distribution calculation; AC = graphite included in calculation; and –GO = coarse alumina coated with graphene oxide.

In order to confirm the results from the mechanical testing, fracture surfaces of the samples before as well as after thermal shock were investigated by SEM. The obtained micrographs are presented in Figure 4, with side-by-side comparisons. Graphite flakes were observed on all surfaces using the BSE signal, as well as alumina grains with different sizes. Smaller dark areas were most likely associated with amorphous carbon generated by the decomposition of the resin binder. However, due to the relatively low carbon yield of phenolic resins, the carbon fraction after coking consisted almost entirely of graphite.

Pores and cracks were also detected, especially in direct proximity of the graphite flakes. This is likely due to graphite having a lower coefficient of thermal expansion than alumina, in particular in the direction parallel to the carbon layers (where it is even negative in some cases). It should be mentioned, however, that such gaps were already present in the coked samples and that the microstructure of all batches was comparable after thermal shock. This is in agreement with the results from the three-point bending tests, which revealed no significant CMOR drop (cf. Figure 3B).

One reason behind the high thermal shock resistance of carbon-bonded alumina (compared to fully dense alumina) is indeed the presence of gaps, which gives flexibility to the material and acts as a damper for the in situ thermal stress generation [15]. It can be expected that additional thermal shock cycles would weaken the microstructure by increasing the total length of the crack network and the width of gaps between graphite and alumina grains.

CMOR values for Al_2_O_3_–C refractories from the literature are compared with the results from the present work in Table 4. Please note the different graphite contents, maximum grain size of the alumina aggregates, as well as different types of additives employed. All samples were produced by pressing, with pressures between 80 and 150 MPa. Also note that most reference compositions already contained at least metallic silicon (usually about 6 wt%), that helps preventing carbon oxidation and resulted in higher CMOR values compared to additive-free reference mixtures. This explains the relatively large CMOR difference with the present results and those by Fruhstorfer et al. [11]. Moreover, a smaller maximum grain size for the alumina grains usually results in higher CMOR values as well [47,52].

## 4. Conclusions

In this phenomenological study, the influence of the particle size distribution and graphene oxide (GO) addition on the physical and mechanical properties of low-carbon (15% graphite) carbon-bonded alumina refractories as well as on their behavior after a single thermal shock cycle was investigated. The following conclusions were obtained:

- Coarse tabular alumina grains can be easily coated using a highly viscous graphene oxide suspension by means of dip coating followed by drying in air.
- The packing density of carbon-bonded refractories increased when the irregularly shaped graphite was considered as matrix component (lubricant) and not as a fraction of the calculated aggregate size distribution with quasi-spherical shape. The resulting higher density led to a lower Young’s modulus *E* and drop of *E* after thermal shock, but nevertheless a high CMOR.
- When the packing density was maximized, the low-carbon carbon-bonded alumina refractories showed high strength values (without GO). On the other hand, if the grain packing was not optimal, the GO addition successfully improved the mechanical properties, likely due to a delamination gap-bridging effect. It can be assumed that, during coking, GO released gases, which caused microcracking in case of a very dense structure, leading to a decrease of CMOR and *E* but also a limited drop of *E* during thermal shock.
- While thermal shock resulted in a (limited) drop of Young’s modulus for all batches, it had no significant impact on the CMOR, regardless of the use of GO in the initial formulation or of the graphite inclusion in the calculation of aggregate size distribution. SEM investigation revealed very similar microstructures in coked as well as thermally shocked samples.

The latter point clearly requires further investigation and will be addressed in an upcoming study. In particular, multiple thermal shock cycles will be applied to confirm and extend the results of the present work. In addition, the effect of different GO additions on the material will be explored. Graphene oxide will be also employed in the production of several type of carbon-bonded refractories in the future. It is evident that further reduction of carbon content in refractories is environmentally friendly and the application of GO could be a technology step towards this target.

## Figures and Tables

**Table 1:** Particle size retention distributions in wt% of the graphites determined by laser granulometry.

Material	Retention Size Class in µm										
	0.1	0.4	1	4	10	40	90	150	315	630	1000
AF 96–97	0	0	0.02	0.48	0.74	2.21	29.71	66.17	0.68	0	0
NFL 92–94	0	0	0.05	1.19	3.64	21.40	26.26	25.75	21.68	0	0

**Table 2:** Batch compositions in wt% (ASD—aggregate size distribution and GO—graphene oxide).

Raw Material	Fraction/Type	Batches Based on Densest ASD *Including* Graphite (AC)		Batches Based on Densest ASD *Excluding* Graphite (A)	
		**No Coating**	**GO-Coating**	**No Coating**	**GO-Coating**
GO-coated alumina	1–3 mm		17.5		12.75
Tabular alumina	1–3 mm	35	17.5	25.5	12.75
	0.5–1 mm	5	5	4.25	4.25
	0–0.5 mm			8.5	8.5
	0–0.2 mm			8.5	8.5
	0–0.02 mm	45	45	38.25	38.25
Graphite	AF 96–97	5	5	5	5
	NFL 92–94	10	10	10	10
Novolac resin *	powder	4	4	4	4
	liquid	2	2	2	2

* As a curing agent, hexamethylene tetramine was used with a content of 10% of the resin amount.

**Table 3:** Means and standard deviations of the bulk and true densities, the open and total porosities, the Young’s modulus *E* and its drop with thermal shock and the cold modulus of rupture (CMOR) in dependence on the graphene oxide addition (–GO), the graphite consideration in the aggregate size distribution calculation (AC = graphite included; A = graphite not included) and the state (1TS—once thermally shocked).

Batch	State	Bulk Density in g/cm${}^{3}$		Open Porosity	True Density	Total Porosity	*E* in GPa	CMOR in	Drop of *E*
		Mass/Dimensions	Immersion	in%	in g/cm${}^{3}$	in%		MPa	in%
AC	coked	2.475 ± 0.019	2.471 ± 0.036	27.17 ± 0.75	3.510 ± 0.008	29.61 ± 1.27	2.52 ± 0.17	1.13 ± 0.12	14.19 ± 2.68
	1TS		2.420 ± 0.053	26.98 ± 1.35	3.488 ± 0.026	30.63 ± 1.34	2.56 ± 0.10	1.08 ± 0.10	
A	coked	2.525 ± 0.011	2.546 ± 0.023	24.19 ± 0.82	3.476 ± 0.007	26.76 ± 0.96	5.44 ± 0.24	2.75 ± 0.27	7.87 ± 0.34
	1TS		2.463 ± 0.072	24.33 ± 1.55	3.462 ± 0.005	28.86 ± 2.45	5.14 ± 0.08	3.00 ± 0.08	
AC–GO	coked	2.406 ± 0.021	2.419 ± 0.034	28.35 ± 1.09	3.474 ± 0.009	30.36 ± 1.09	4.83 ± 0.40	1.63 ± 0.16	4.92 ± 0.94
	1TS		2.257 ± 0.209	26.98 ± 3.89	3.465 ± 0.011	34.88 ± 7.22	4.92 ± 0.12	1.66 ± 0.12	
A–GO	coked	2.424 ± 0.016	2.442 ± 0.013	28.67 ± 0.67	3.510 ± 0.006	30.42 ± 0.42	3.48 ± 0.06	1.25 ± 0.02	6.62 ± 1.99
	1TS		2.420 ± 0.008	28.49 ± 0.88	3.467 ± 0.009	30.21 ± 0.43	3.68 ± 0.05	1.40 ± 0.10	

**Table 4:** Comparison of literature works on reinforcement of carbon-bonded alumina with the present study. Coking temperature = 1000 °C; CNTs = Carbon nanotubes; ANs = Alumina nanosheets; GONs = Graphene oxide nanosheets; and n–Si = n-doped (with phosphorous) silicon, semiconductor.

Reference Composition				With Extra Additive				Ref.
Graphite Content	Max. Grain Size	Additives	CMOR (MPa)	Additive Type	Additive Amount (wt%)	CMOR (MPa)	ΔCMOR (%)	
30%	0.60 mm	Si	15.9					
20%	0.60 mm	Si	12.6	Spinel	0.1	14.2	+12.7	[52]
				ANs	0.1	15.5	+23.0	
				CNTs (China)	0.3	14.9	+18.3	
				CNTs (Germany)	0.3	13.3	+5.5	
				Spinel, ANs	0.1/0.1	11.3	−10.3	
				Spinel, CNTs (China)	0.1/0.3	14.3	+13.4	
				ANs, CNTs (China)	0.1/0.3	12.9	+2.4	
20%	0.60 mm	Si	10.31	n–Si	0.5	13.20	+28.0	[47]
				n–Si, CNTs, ANs	0.5/0.3/0.1	14.51	+40.7	
				CNTs, ANs	0.3/0.1	12.56	+21.8	
20%	0.60 mm	Si	6.60	CNTs, ANs, n-Si	0.3/0.1/0.5	6.14	−7.0	[27]
				n–Si	0.5	6.80	+3.0	
				CNTs, ANs	0.3/0.1	6.63	+0.5	
15%	3 mm		1.13 (AC)	Graphene oxide	0.4	1.63	+44.2	This work
			2.75 (A)			1.25	−54.5	
1%	3 mm	Al, Si, microsilica	12.95	Alumina-coated graphite	0.5 *	11.80	−8.8	[28]
					1 *	9.94	−23.2	
1%	2 mm	Al, Si, microsilica	9.09	CNTs	0.05	12.08	+32.9	[60]
					0.1	10.66	+17.3	
					0.3	10.08	+10.9	
					0.5	9.94	+9.4	
					1	7.77	−14.5	
1%	2 mm	Al, Si, microsilica	7.30	GONs	0.1	12.22	+67.4	[42]
					0.21	12.88	+76.4	
					0.55	11.41	+56.3	
					0.88	11.29	+54.7	
0% (resin-bonded)	3 mm		2.38	CNTs, ANs	0.1/0.3	3.81	+60.1	[11]

* = amount of additive replacing graphite.

## Data Availability

Not applicable.
